# Nuclear Factor-κB–Dependent Epithelial to Mesenchymal Transition Induced by HIF-1α Activation in Pancreatic Cancer Cells under Hypoxic Conditions

**DOI:** 10.1371/journal.pone.0023752

**Published:** 2011-08-22

**Authors:** Zhuo-Xin Cheng, Bei Sun, Shuang-Jia Wang, Yue Gao, Ying-Mei Zhang, Hao-Xin Zhou, Guang Jia, Yong-Wei Wang, Rui Kong, Shang-Ha Pan, Dong-Bo Xue, Hong-Chi Jiang, Xue-Wei Bai

**Affiliations:** 1 Department of Pancreatic and Biliary Surgery, The First Affiliated Hospital of Harbin Medical University, Harbin, People's Republic of China; 2 Department of Surgery, University Hospitals, Case Western Reserve University, Cleveland, Ohio, United States of America; 3 Central Laboratory, The First Affiliated Hospital of Harbin Medical University, Harbin, People's Republic of China; University of Nebraska Medical Center, United States of America

## Abstract

**Background:**

Epithelial to mesenchymal transition (EMT) induced by hypoxia is one of the critical causes of treatment failure in different types of human cancers. NF-κB is closely involved in the progression of EMT. Compared with HIF-1α, the correlation between NF-κB and EMT during hypoxia has been less studied, and although the phenomenon was observed in the past, the molecular mechanisms involved remained unclear.

**Methodology/Principal Findings:**

Here, we report that hypoxia or overexpression of hypoxia-inducible factor-1α (HIF-1α) promotes EMT in pancreatic cancer cells. On molecular or pharmacologic inhibition of NF-κB, hypoxic cells regained expression of E-cadherin, lost expression of N-cadherin, and attenuated their highly invasive and drug-resistant phenotype. Introducing a pcDNA3.0/HIF-1α into pancreatic cancer cells under normoxic conditions heightened NF-κB activity, phenocopying EMT effects produced by hypoxia. Conversely, inhibiting the heightened NF-κB activity in this setting attenuated the EMT phenotype.

**Conclusions/Significance:**

These results suggest that hypoxia or overexpression of HIF-1α induces the EMT that is largely dependent on NF-κB in pancreatic cancer cells.

## Introduction

Pancreatic cancer, which is one of the most aggressive and lethal cancers worldwide, is highly resistant to chemotherapy [1]. Even systemic therapy with gemcitabine, a current first-line treatment for advanced pancreatic cancer offers only modest benefit due to intrinsic or acquired chemoresistance [2], [3]. Further, recent clinical studies indicate that only 12% of patients with advanced pancreatic cancer have a response to gemcitabine [4]. The poor response rate suggests that pancreatic cancer either rapidly develops or has gemcitabine chemoresistance. The mechanisms by which chemoresistance arises in pancreatic cancer are unknown; thus a better understanding of how resistance arises and what molecular alterations cause or correlate with resistance is likely to lead to novel therapeutic strategies for pancreatic cancer.

Hypoxia is an environmental stimulus that plays a key role in development and cancer progression. Tumoral hypoxia or expression HIF-1 (hypoxia-inducible factor-1) has been linked to an aggressive phenotype which correlates with a poor response to chemotherapy and a worse overall survival of cancer patients [5], [6]. HIF-1 is a heterodimeric protein consisting of HIF-1β, a constitutively expressed subunit, and HIF-1α, an oxygen-sensitive inducible subunit. Under normoxic conditions, HIF-1α protein is hydroxylated by a family of oxygen-dependent prolyl hydroxylases (PHD1–3); this targets it for polyubiquitination by a protein complex containing von Hippel-Lindau protein (pVHL) and then degradation. Under hypoxic conditions, prolyl hydroxylases are inactivated, and HIF-1α degradation is blocked; this allows HIF-1α to accumulate and associate with HIF-1β to form a functional transcription complex that triggers the transcription of a host of hypoxia-inducible genes [7].

Epithelial to mesenchymal transition (EMT) is the process by which adherent epithelial cells convert to motile mesenchymal cells and is essential in embryonic development. EMT is now known to also occur in a variety of diseases including the progression of cancer [8]. In the recent study, evidence is provided suggesting that moderate hypoxic conditions can trigger, as an independent factor, an EMT programme leading different human cancer cells to significantly increase invasiveness [9]. Meanwhile, some studies also reported that activation of NF-κB is closely involved in the progression of EMT [10]–[13]. Detailed examinations of the multiple facets of the EMT program have revealed its involvement in more than just invasion and metastasis; recent studies showed that the phenotype of EMT is associated with chemoresistance in diverse solid tumors [14]–[17].

Nuclear factor-kappa B (NF-κB) represents a family of transcription factors that modulate expression of genes with diverse functions. The activity of NF-κB is regulated by the NF-κB inhibitory protein (IκB), that binds to and sequesters NF-κB family members in the cytoplasm. When the NF-κB pathway is activated, IκB is phosphorylated by IκB kinase (IKK), which phosphorylates IκB. Phosphorylated IκB is subjected to ubiquitination and proteasome-mediated degradation, which results in the translocation of NF-κB to the nucleus. NF-κB is a ubiquitous transcription factor regulated by many stimuli including hypoxia, cytokines and chemotherapeutic drugs, and has recently emerged as a target for cancer. NF-κB is constitutively activated in most human pancreatic cancer cells and primary tumor specimens, but not in normal pancreatic tissues or nontumorigenic cell lines [18]–[20]. Some recent studies showed that hypoxia can activate NF-κB and induce resistance of pancreatic cancer cells to gemcitabine [21]–[23]. Previously, we reported that using Dihydroartemisinin or small interfering RNA (siRNA) inactivates NF-κB and potentiates the antitumor effect of gemcitabine on pancreatic cancer both in vitro and in vivo [24], [25].

In this study, we sought evidences that pancreatic cancer cells (PANC-1, BxPC3) under hypoxic conditions undergo the process of EMT and acquire invasive and drug-resistant phenotypes in a NF-κB–dependent fashion. Herein, we demonstrated that hypoxia or overexpression of HIF-1α activated NF-κB and promoted EMT in pancreatic cancer cells. On molecular or pharmacologic inhibition of NF-κB, hypoxic cells regained expression of E-cadherin, lost expression of N-cadherin, and attenuated their highly invasive and drug-resistant phenotype. Introducing a pcDNA3.0/HIF-1α into pancreatic cancer cells under normoxic conditions heightened NF-κB activity, phenocopying EMT effects produced by hypoxia. Conversely, inhibiting the heightened NF-κB activity in this setting reversed the EMT phenotype. Taken together, these results suggest that hypoxia or overexpression of HIF-1α induce the EMT that is largely dependent on NF-κB in pancreatic cancer cells.

## Results

### Hypoxia results in the morphologic and cell biological changes characteristic of EMT in pancreatic cancer cells

**Figure 1:**
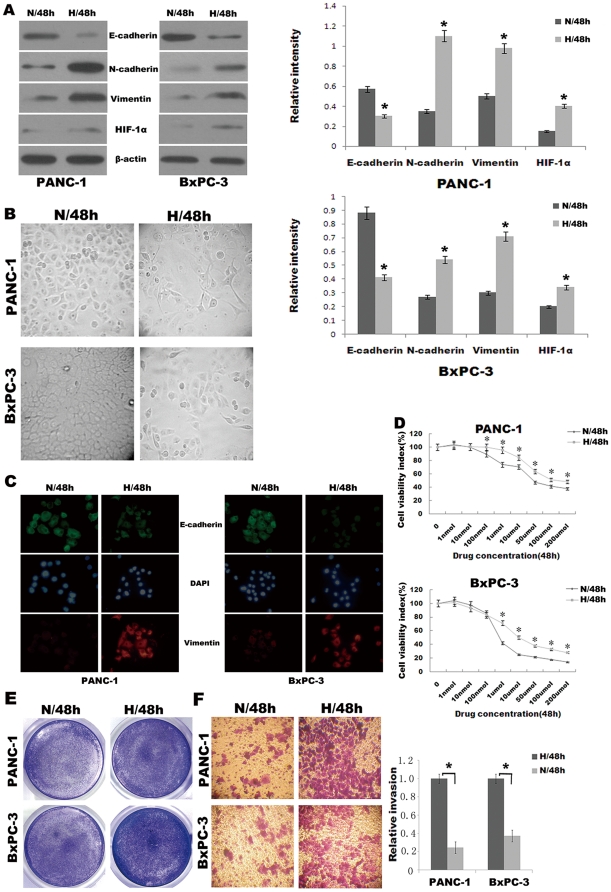
Hypoxia induces EMT phenotype in pancreatic cancer cells. (A) Western blot analysis of epithelial marker (E-cadherin) and mesenchymal markers (Vimentin, N-cadherin) and HIF-1α of PANC-1, BxPC-3 cells under normoxic (N) or hypoxic (H) conditions for 48 h. A representative blot from three independent experiments was shown. The histogram showed the average volume density corrected for the loading control (β-actin). *, *p*<0.05. (B) Phase contrast analysis (original magnification, ×100) of morphological changes detected in pancreatic cancer cells under normoxic or hypoxic conditions for 48 h. (C) Immuofluorescence staining of E-cadherin and Vimentin in PANC-1, BxPC-3 cells under normoxic or hypoxic conditions for 48 h. Green represents E-cadherin staining, whereas red represents Vimentin staining. Blue signal represents nuclear DNA staining by DAPI. (D) The viability of cells was assessed by the Cell Counting Kit-8 assay and used to calculate the viability index. PANC-1 and BxPC-3 cells were exposed to gemcitabine (0 µmol/L) for 48 h as the baseline. *, *p*<0.05. (E) The proliferation of cells was measured by crystal violet assay. Figures were selected as representative scenes from three independent experiments. (F) Matrigel invasion assay. Left, photomicrographs of cells that have passed through Matrigel under normoxic or hypoxic conditions for 48 h (original magnification, ×100). Right, quantification of invasion. *, *p*<0.05.

To recapitulate the effects of hypoxia as it occurs in pancreatic cancer, we exposed 55–60% subconfluent pancreatic cancer cells (PANC-1, BxPC-3) to hypoxic conditions (1% O_2_, 5% CO_2_, and 94% N_2_) up to 48 h. We observed marked differences in the morphology and tendency to form cellular nests or clusters between the pancreatic cancer cells under hypoxic conditions and their normoxic counterparts (95% air and 5% CO_2_). The normoxic cells exhibited a polygonal shape and cobblestone-like sheets, indicative of an epithelial phenotype. In contrast, the hypoxic cells started to loose cell contacts, scattered from cell clusters and acquired a elongated, fusiform morphology with dendritic processes, consistent with a mesenchymal transition (Fig. 1B).

To confirm whether pancreatic cancer cells underwent EMT exposed to hypoxia, we also determined the expression of markers of epithelial and mesenchymal phenotypes by Western blot analysis. As shown in Fig. 1A, hypoxic cells underwent a “cadherin switch,” whereby they manifested reduced E-cadherin expression and increased N-cadherin expression. In addition, the expression of HIF-1α, an important marker of hypoxia pathway, was found intensely expressed under hypoxic condition for 48 h (Fig. 1A). Immunofluorescence staining was employed to further confirm EMT phenotype changes of pancreatic cancer cells under hypoxic condition. It was shown that the expression of E-cadherin decreased and the expression of Vimentin increased when compared with those in normoxic cells (Fig. 1C).

Cancer cells that have undergone EMT tend to exhibit greater drug resistance and invasiveness. As such, we examined these properties in normoxic cells versus hypoxic cells in Cell Counting Kit-8 assay, Crystal violet assay and Matrigel invasion assay. Striking differences were observed. Cell Counting Kit-8 assay was used to assess the cell viability rate. PANC-1 and BxPC-3 cells were exposed to gradient doses of gemcitabine (0–200 µmol/L) for 48 h. Data are shown in Fig. 1D, when treated with concentration of gemcitabine larger than 100 nmol/L, hypoxic cells showed significantly higher cell viability rate than the normoxic cells. These results were further confirmed by Crystal violet assay (Fig. 1E), when cells were treated with gemcitabine (10 µmol/L for PANC-1 and 500 nmol/L for BxPC-3) under hypoxic or normoxic conditions for 48 h, hypoxic cells showed significantly higher cell viability rate than normoxic cells. Matrigel invasion assay was used to assess the cell invasiveness. Both PANC-1 and BxPC-3 cells under hypoxic conditions for 48 h readily migrated through the Matrigel chamber in relatively high numbers, whereas their normoxic partners exhibited a marked reduction in invasion (Fig. 1F).

Taken together, our data indicating changes in morphology, growth pattern, protein expression, drug resistance and invasion support the notion that hypoxia leads to EMT in pancreatic cancer cells.

### Pancreatic cancer cells under hypoxic conditions exhibit heightened NF-κB activity

**Figure 2:**
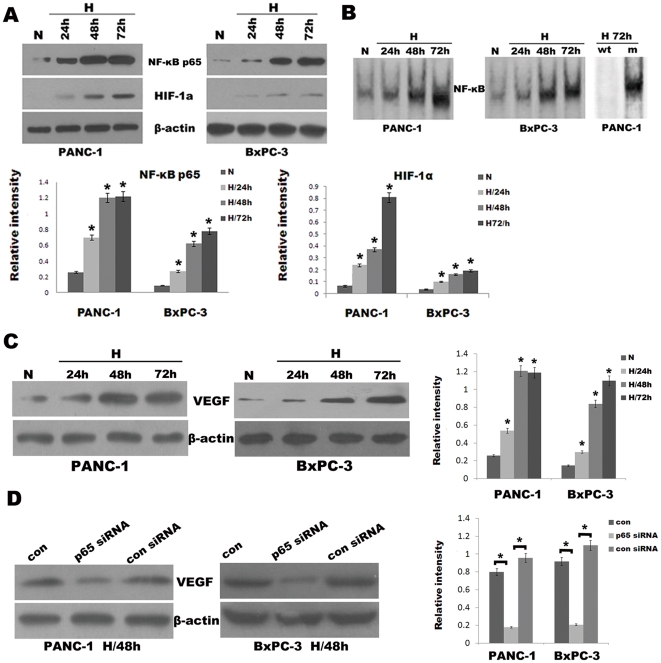
Hypoxia induced HIF-1α and NF-κB overexpression in pancreatic cancer cells. PANC-1 and BxPC-3 cells were cultured under normoxic (N) or hypoxic conditions (H) for various periods (24 h, 48 h, 72 h). After each indicated incubation period, the cells were collected. Nuclear extracts and total protein extracts were prepared. (A) NF-κB p65 and HIF-1α were analyzed by Western blot analysis from respective cell homogenate. The histogram showed the average volume density corrected for the loading control (β-actin). *, *p*<0.05. (B) EMSA for NF-κB DNA-binding activity on respective nuclear extracts. Competitive assay confirmed the specificity of NF-κB binding to the DNA (see Materials and methods for details). Right two lanes, wt, wild-type; m, mutant. (C) VEGF was analyzed by Western blot analysis from respective cell homogenate. The histogram showed the average volume density corrected for the loading control (β-actin). *, *p*<0.05. (D) Western blot analysis. Effects of the NF-κB p65 siRNA on VEGF expression of PANC-1 and BxPC-3 cells under hypoxic conditions for 48 h. The histogram showed the average volume density corrected for the loading control (β-actin). *, *p*<0.05.

Some studies have previously shown that hypoxia results in activation of NF-κB [19], [20]. Thus, we sought to determine whether the EMT observed in pancreatic cancer cells under hypoxic conditions was attributable to heightened NF-κB activity. First, we documented that NF-κB p65 proteins and NF-κB DNA binding activity were indeed increased in hypoxic pancreatic cancer cells compared with the normoxic counterparts as determined by Western blot analysis and EMSA. Meanwhile, HIF-1α was also analyzed by Western blot analysis (Fig. 2A). PANC-1 and BxPC-3 cells were incubated under hypoxic conditions for different periods of time (24 h, 48 h, 72 h). After each indicated incubation period, the cells were collected, and total or nuclear proteins were extracted. As shown in Fig. 2A and B, NF-κB p65 proteins and NF-κB DNA binding activity were increased 24 h after initiation of hypoxia and maintain high levels. The specificity of gel-shifted bands in the EMSA was documented by cold competition experiments, in which excess cold wild-type but not cold mutant κB probe abrogated the signals from the shifted bands.

Meanwhile, HIF-1α was also analyzed by Western blot analysis. As shown by Western blotting (Fig. 2A), HIF-1α protein levels in pancreatic cancer cells were up-regulated during hypoxia. The increase in HIF-1α protein level was possibly due to protein stability effects as studied previously [7]. Since the expression of HIF-1α target gene VEGF, a growth factor that can promote EMT, is mediated by NF-κB, we also determine whether hypoxia stimulates VEGF expression. As shown in Fig. 2C, Western blotting showed that VEGF protein expression increased significantly after 24 h of hypoxia and maintained high levels. By contrast, the up-regulation of VEGF was suppressed by silencing NF-κB p65 expression in hypoxic pancreatic cancer cells using the NF-κB p65-specific siRNA, while no effect was seen in cells transfected with the control siRNA (Fig. 2D).

### Inhibition of NF-κB activity in pancreatic cancer cells under hypoxic conditions mediates EMT

**Figure 3:**
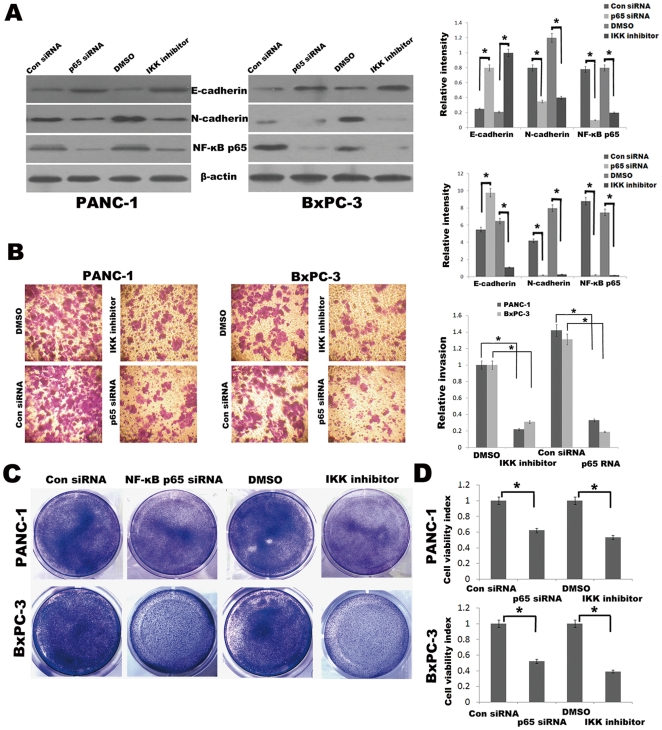
Inhibition of NF-κB reverses EMT characteristics in pancreatic cancer cells under hypoxic conditions. (A) Western blot analysis. Effects of the NF-κB p65 siRNA and IKK inhibitor BAY 11-7082 on N-cadherin and E-cadherin expression of PANC-1 and BxPC-3 cells under hypoxic conditions for 48 h. The histogram showed the average volume density corrected for the loading control (β-actin). *, *p*<0.05. (B) Matrigel invasion assays. Left, photomicrographs after cells were treated with NF-κB p65 siRNA or BAY 11-7082 (10 µmol/L) or appropriate respective controls. Original magnification, ×100. Right, quantification of invasion assay. *, *p*<0.05. (C) The proliferation of cells was measured by Crystal violet assay. Figures were selected as representative scenes from three independent experiments. (D) The viability of cells was assessed by the Cell Counting Kit-8 assay and used to calculate the viability index. *, *p*<0.05.

Having established that hypoxia results in elevated NF-κB activity in pancreatic cancer cells, we next investigated the potential for inhibition of NF-κB to attenuate the mesenchymal characteristics of hypoxic cells. Toward this end, we used both molecular and pharmacologic means of inhibiting NF-κB in these hypoxic cells and then compared the resulting phenotype with control-treated cells. Molecular inhibition was accomplished by siRNA to downregulate the p65 subunit of NF-κB. We also used a commercially available IKK inhibitor BAY 11-7082 to pharmacologically block NF-κB activity. Inhibition of NF-κB activity by either the NF-κB p65 siRNA or BAY 11-7082 under hypoxic conditions for 48 h resulted in a change in protein expression characterized by increased E-cadherin and reduced N-cadherin expression as determined by Western blot analysis (Fig. 3A), consistent with reversion to an epithelial phenotype. Importantly, inhibition of NF-κB by either NF-κB p65 siRNA or BAY 11-7082 led to a marked decrease in the invasiveness of hypoxic cells compared with control treated cells in Matrigel invasion assay under hypoxic conditions for 48 h (Fig. 3B). When cells were treated with gemcitabine (10 µmol/L for PANC-1 and 500 nmol/L for BxPC-3) for 48 h with inhibition of NF-κB activity by either NF-κB p65 siRNA or IKK inhibitor under hypoxic conditions, they also showed significantly lower viability rate than their respective controls in Crystal violet assay (Fig. 3C) and Cell Counting Kit-8 assay (Fig. 3D). Despite these changes in protein expression, invasiveness and drug resistance attributable to NF-κB blockade, we did not observe any profound changes in the morphology or growth patterns of pancreatic cancer cells under hypoxic conditions, an observation that implicates NF-κB–independent biochemical events that also contribute to the EMT phenotype in hypoxic pancreatic cancer cells.

### Pancreatic cancer cells under hypoxic conditions exhibit NF-κB–dependent upregulation of Twist, transcriptional regulators of the EMT program

**Figure 4:**
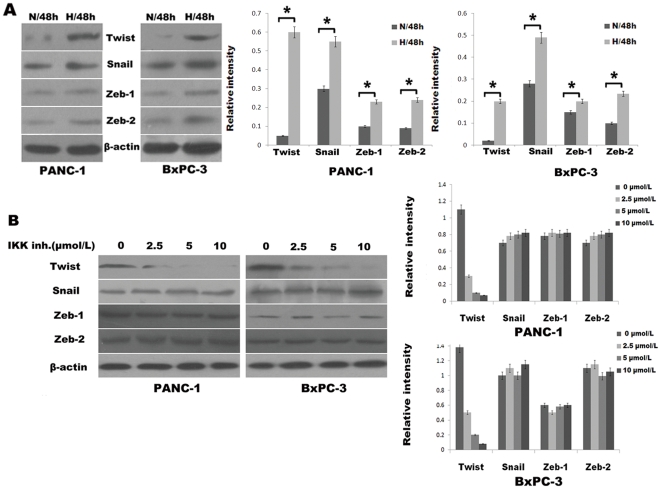
Heightened expression of Twist in pancreatic cancer cells under hypoxic conditions is attributable to increased NF-κB activity. (A) Western blot analysis of baseline expression of EMT-mediating transcription factors: Twist, Snail, Zeb1, and Zeb2 in pancreatic cancer cells under normoxic (N) versus hypoxic (H) conditions for 48 h. The histogram showed the average volume density corrected for the loading control (β-actin). *, *p*<0.05. (B) Western blot analysis. Dose-dependent effects of a 48-h exposure of the IKK inhibitor BAY 11-7082 on expression of Twist, Snail, Zeb1, and Zeb2 under hypoxic conditions. A representative blot from three independent experiments was shown. The histogram showed the average volume density corrected for the loading control (β-actin).

The gene expression program that mediates EMT is regulated by one or more transcription factors, including Twist, Zeb1, Zeb2, and Snail [26]. These transcription factors influence the expression of cadherins and metalloproteinases, among other proteins involved in EMT. That these transcription factors are transcriptionally induced by upstream signaling pathways, including NF-κB [26], prompted us to observe their differential expression in pancreatic cancer cells under hypoxic conditions versus normoxic conditions by Western blot analysis. Hypoxic pancreatic cancer cells exhibited substantial upregulation of Twist, Snail, Zeb1, and Zeb2 as compared with normoxic cells (Fig. 4A). Pharmacologic inhibition of IKK by BAY 11-7082 in hypoxic cells resulted in a dose-dependent decrease in Twist expression but no notable changes in Zeb1, Zeb2 or Snail expression, a finding that indicts the heightened state of NF-κB as an etiologic biochemical force underlying Twist overexpression in pancreatic cancer cells under hypoxic conditions (Fig. 4B). These findings indicate that the augmented expression of Twist that occurs in the setting of pancreatic cancer cells under hypoxic conditions is mediated by heightened NF-κB activity.

### Overexpression of HIF-1α in pancreatic cancer cells under normoxic conditions induces EMT in NF-κB–dependent manner

**Figure 5:**
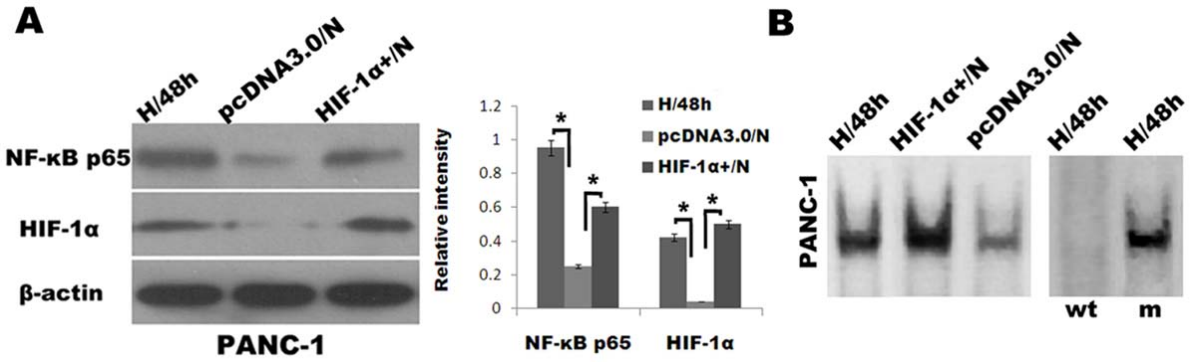
Overexpression of HIF-1α in pancreatic cancer cells under normoxic conditions induces NF-κB activation. (A) Western blot analysis of NF-κB p65 and HIF-1α expression of PANC-1 cells under hypoxic conditions for 48 h (H/48 h), PANC-1 cells transduced with pcDNA3.0 empty vector (pcDNA3.0/N) and pcDNA3.0/HIF-1α (HIF-1α^+^/N) under normoxic conditions for 48 h. The histogram showed the average volume density corrected for the loading control (β-actin). *, *p*<0.05. (B) EMSAs for NF-κB DNA-binding activity in H/48 h, HIF-1α^+^/N, and pcDNA3.0/N PANC-1 cells. Right two lanes, cold competition EMSA.

Recent reports have established an etiologic link between HIF-1α expression to suppression of E-cadherin and acquisition of a mesenchymal phenotype [27]. Thus, we hypothesized that the EMT that pancreatic cancer cells undergo in response to HIF-1α expression occurs, at least in part, due to the HIF-1α-mediated activation of the NF-κB pathway. First, we transiently introduced a pcDNA3.0/HIF-1α into PANC-1 under normoxic conditions (HIF-1α^+^/N) using a LipofectamineTM 2000. Whereas HIF-1α was almost undetectable in PANC-1 under normoxic conditions transduced with control empty vector (pcDNA3.0/N), the HIF-1α levels in HIF-1α^+^/N cells were comparable with those in the cells under hypoxic conditions for 48 h (Fig. 5A). Next, we confirmed that NF-κB activity did in fact increase in these HIF-1α^+^/N cells than pcDNA3.0/N cells. Indeed, HIF-1α^+^/N cells exhibited heightened NF-κB activity as determined by Western blot analysis and EMSA (Fig. 5A and B).

**Figure 6:**
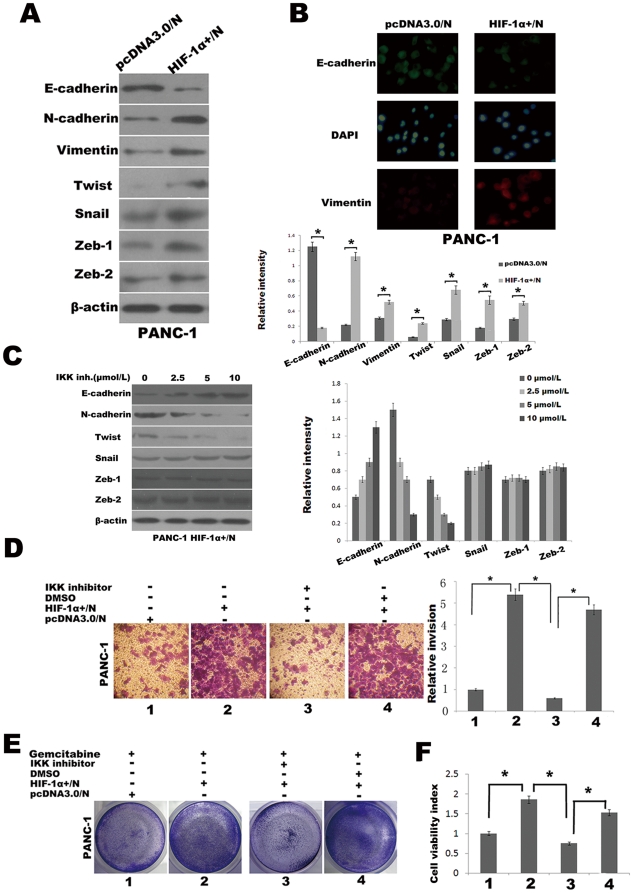
NF-κB blockade reverses the EMT driven by the HIF-1α. (A) Western blot analysis of levels of indicated proteins in PANC-1 cells transduced with pcDNA3.0 empty vector (pcDNA3.0/N) and pcDNA3.0/HIF-1α (HIF-1α^+^/N) under normoxic conditions for 48 h. The histogram showed the average volume density corrected for the loading control (β-actin). *, *p*<0.05. (B) EMT phenotype changes was confirmed with Immuofluorescence microscopy staining of E-cadherin and Vimentin. Green represents E-cadherin staining, whereas red represents Vimentin staining. Blue signal represents nuclear DNA staining by DAPI. (C) Western blot analysis. Dose-dependent effects of a 48-h exposure to the IKK inhibitor BAY 11-7082 on the indicated proteins. The histogram showed the average volume density corrected for the loading control (β-actin). (D) Matrigel invasion assays. Effects of a 48-h BAY 11-7082 (10 µmol/L) on invasion of PANC-1 cells under normoxic conditions. Left, photomicrographs at original magnification of ×100; Right, histogram to illustrate invasion assay results. *, *p*<0.05. (E) Crystal violet assay. Effects of a 48-h BAY 11-7082 (10 µmol/L) on gemcitabine susceptibility of PANC-1 cells under normoxic conditions. Figures were selected as representative scenes from three independent experiments. (F) The Effects of IKK inhibitor on gemcitabine susceptibility of PANC-1 cells under normoxic conditions was also measured by Cell Counting Kit-8 assay and used to calculate the viability index. *, *p*<0.05.

Having established that HIF-1α^+^/N cells manifest heightened NF-κB activity, we next assessed these cells for evidence of EMT. Compared with pcDNA3.0/N cells, HIF-1α^+^/N cells exhibited an increase in N-cadherin expression and suppression of E-cadherin under normoxic conditions for 48 h as determined by Western blot analysis (Fig. 6A). Immunofluorescence staining was employed to further confirm an EMT induced by HIF-1α under normoxic conditions, the expression of E-cadherin decreased and Vimentin increased in the HIF-1α^+^/N cells compared with pcDNA3.0/N cells under normoxic conditions for 48 h (Fig. 6B). This cadherin switch was also associated with increased Twist, Snail, Zeb1, and Zeb2 expression (Fig. 6A), findings that are reminiscent of those observed in pancreatic cancer cells under hypoxic conditions (Fig. 4A). The role of NF-κB in modulating these protein expression changes was illustrated by the effects of inhibition of NF-κB in HIF-1α^+^/N cells by exposure to the IKK inhibitor BAY 11-7082 (0–10 µmol/L), which reversed the cadherin switching phenomenon as well as reduced the expression of Twist in a dose-dependent but no notable changes in Zeb1, Zeb2 or Snail expression (Fig. 6C). Invasion and gemcitabine resistance of HIF-1α^+^/N cells and its dependence on heightened NF-κB activity were evaluated in Matrigel invasion assay, Crystal violet assay and Cell Counting Kit-8 assay. For example, invasiveness of HIF-1α^+^/N cells through a Matrigel chamber was significantly greater than that of pcDNA3.0/N cells under normoxic conditions for 48 h (Fig. 6D). Similarly, when cells were treated with gemcitabine (10 µmol/L) for 48 h under normoxic conditions, significantly higher cell viability rate was showed in HIF-1α^+^/N cells compared with pcDNA3.0/N cells as determined by Crystal violet assay (Fig. 6E) and Cell Counting Kit-8 assay (Fig. 6F). The enhanced invasion and gemcitabine resistance of HIF-1α^+^/N cells were abrogated by NF-κB blockade by means of exposure to the IKK inhibitor BAY 11-7082 (10 µmol/L) under normoxic conditions for 48 h (Fig. 6D, E and F). Thus, the augmentation in invasiveness and gemcitabine resistance in PANC-1 that are typified by hypoxia occurs as a result of activation of the NF-κB pathway induced by HIF-1α.

## Discussion

In recent years, it has become increasingly clear that EMT plays important roles in the progression of cancer and is also responsible for the resistant phenotype of cancer cells to conventional chemotherapeutics [8]. Various factors, including hypoxia, have been indicted as inducing this phenomenon through induction of Twist, Snail, Zeb1, and Zeb2 depending on the cellular contexts [11], [26]–[28]. A hypoxic microenvironment is commonly found in the central region of solid tumors. The connection between hypoxia and EMT has been previously reported, and HIF-1α has been indicted as mediating this phenomenon. However, the molecular underpinnings of HIF-1α-induced up regulation of these EMT-inducing transcription factors have been largely undefined, although evidence in support of the ability of HIF-1α to directly induce Twist transcription has been recently described in human embryonic kidney cells [27].

Previous study showed that hypoxia lead to NF-κB activation and such activation was mediated by phosphorylation of IκBα on tyrosine residues [28]. NF-κB activation is a critical component in the transcriptional response to hypoxia. Our data presented herein indicated that NF-κB activation is increased under hypoxic conditions as well as transiently transfected with a pcDNA3.0/HIF-1α in pancreatic cancer cells under normoxic conditions. Meanwhile, the heightened NF-κB activity under hypoxia is inhibited by IKK inhibitor BAY-11-7082, which can inhibit the phosphorylation and degradation of IκBα, and prevents the translocation of p65/p50. Both our data and previous studies showed that biochemical pathways leading to NF-κB activation converged on the IKK complex under hypoxic conditions. Recently, it was reported that NF-κB transcriptionally induces HIF-1α expression in murine macrophages, liver, and brain [29]. We also found that pharmacologic inhibition of NF-κB, IKK inhibitor BAY-11-7082, resulted in reduced HIF-1α expression in both PANC-1 and BxPC-3 cells under hypoxic conditions (data not shown). These findings suggest the existence of a biochemical loop, whereby HIF-1α activates NF-κB and vice versa. Interrupting this loop in pancreatic cancer cells at one or more of the many biochemical steps that link HIF-1α to NF-κB is apt to have a significant effect on pancreatic cancer growth. However, the relationship between HIF-1α and NF-κB in the process of tumor development is particularly complex and requires further exploration. Furthermore, we have shown that the EMT program attributable to hypoxia or overexpression of HIF-1α is largly driven by activation of the classic NF-κB pathway. This EMT program is characterized by Vimentin and N-cadherin expression and E-cadherin suppression, striking morphologic changes, a highly invasive and gemcitabine-resistant mesenchymal phenotype.

The recent study showed that the expression of HIF-1α target gene VEGF, a growth factor that can promote EMT in prostate cancer [30]. In this study, the data also showed that protein expression of VEGF increased significantly in pancreatic cancer cells under hypoxic conditions and the up-regulation of VEGF, induced by hypoxia, was abolished by NF-κB p65 siRNA. We also found that these cells incubated under hypoxic conditions or transfected with pcDNA3.0/HIF-1α under normoxic conditions exhibit heightened NF-κB activity, the inhibition of which results in reversion of the cadherin switch to that of an epithelial phenotype typified by reduced invasion and increased gemcitabine sensitivity, findings that implicate NF-κB as a principal mediator of the HIF-1α-induced EMT program in pancreatic cancer cells under hypoxic conditions.

Other studies have previously identified Zeb1, Zeb2, and Snail as central regulators of E-cadherin suppression and EMT in pancreatic cancer cells [11], [31]. However, the expression of Twist was not detected under normoxic conditions and the Twist gene was activated after exposure to hypoxia in five pancreatic cancer cell lines [32]. In this study, we showed that the EMT induced by hypoxia or overexpression of HIF-1α was associated with increased Twist, Snail, Zeb1, or Zeb2 expression. We also found that pharmacologic inhibition of IKK by BAY 11-7082 in hypoxic cells resulted in a dose-dependent decrease in Twist expression but no notable changes in Zeb1, Zeb2 or Snail expression, which suggests that the specific transcription factors regulating EMT depend on the cellular context and the specific state of cells. This mesenchymal transformation can in large part be attenuated by inhibiting NF-κB through either molecular or pharmacologic approaches, although NF-κB blockade does not promote a complete reversion to an epithelial phenotype, as evidenced by maintenance of the Snail, Zeb1, or Zeb2 expression in the face of NF-κB p65 siRNA or IKK inhibition. This latter finding suggests the existence of NF-κB–independent EMT regulatory pathways in pancreatic cancer cells under hypoxic conditions that remain to be defined. In view of all of these EMT-modulating transcription factors, including Twist, Snail, Zeb1, and Zeb2, can be modulated by NF-κB under some specific conditions [26], so that it is plausible that the NF-κB activation resulting from hypoxia or overexpression of HIF-1α represents a unifying biochemical event that accounts for the EMT observed in pancreatic cancer cells.

In conclusion, these results should provide incentives for further investigation and optimization in establishing the mechanistic role of HIF-1α and NF-κB in the attenuation of EMT characteristics and drug resistance and their utility in the clinical setting for the treatment of pancreatic cancer for which there is no effective and curative therapy.

## Materials and Methods

### Cell lines and reagents

The human pancreatic cancer cell lines BxPC-3 and PANC-1 were obtained from the American Type Culture Collection (Rockville, Maryland, USA). Both cell lines were routinely cultured at 37°C in RPMI 1640 medium supplemented with 10% fetal bovine serum, penicillin (100 U/ml) and streptomycin (100 µg/ml) in an incubator with 95% air and 5% CO_2_. Hypoxic cells were incubated in the same conditions but in a hypoxic incubator (Precision Scientific, Winchester, VA, USA) with 1% O_2_, 5% CO_2_, and 94% N_2_. The IKK inhibitor BAY 11-7082 was purchased from Merck Biosciences and diluted in culture medium to obtain the desired concentration. The antibodies against NF-κB p65, Snail (H-130), Twist (H-81), Zeb-1, Zeb2 and β-actin were purchased from Santa Cruz Biotechnology Inc., CA, USA. The HIF-1α Ab was purchased from Abcam Inc., MA, USA. The Vimentin, E-cadherin and N-cadherin Ab were purchased from ZSGB-BIO Inc., Beijing, China. Gemcitabine was purchased from Lily France, Fegersheim, France. Cell Counting Kit-8 (Dojin Laboratory, Kumamoto, Japan). Endofree Plasmid Maxi Kit (Qiagen, chatsworth, CA). LipofectamineTM 2000 and Plus reagent (Invitrogen).

### Assessment of cellular morphological changes and determination of cell viability

Cellular morphology was evaluated using phase-contrast microscopy, and photographs were captured with a digital camera (Canon Power shot A640). Cell viability was determined by the Cell Counting Kit-8 assay and Crystal violet assay. a) Cell Counting Kit-8 assay: BxPC-3 (4×10^3^/well) and PANC-1 (3×10^3^/well) cells were split into 96-well plates and incubated overnight in a humidified CO_2_ incubator (95% air and 5% CO_2_) to allow the cells to adhere and assume a healthy condition, and then exposed to gradient doses of gemcitabine (0–200 µmol/L) for 48 h under hypoxic or normoxic conditions. The cells were then incubated with WST-8 solution at 37°C for 1 h and the absorbance at 450 nm was measured on a microplate reader (MPR-A4i, Tosoh Corporation, Tokyo, Japan). The cells cultured in RPMI 1640 medium served as the control. The cell viability index was calculated according to the formula: experimental OD value/control OD value×100%. All experiments were done in triplicate and repeated thrice. Two cell lines underwent the same procedure. b) Crystal violet assay: cells (5×10^4^/well) were seeded into 6-well plates and cultured overnight. Then the medium was replaced with complete culture medium containing gemcitabine (10 µmol/L for PANC-1 and 500 nmol/L for BxPC-3) for additional 48 h under hypoxic or normoxic conditions. Cells were then washed twice with pre-warmed PBS, and then cells remaining were stained for 1 h with a crystal violet solution (0.1% crystal violet, 20% methanol). After removal of the crystal violet solution, the plates were washed three times by immersion in a beaker filled with tap water. Plates were left to dry at 37°C, and then the images were photographed by camera.

### Matrigel invasion assay

The Matrigel invasion assay was performed according to the manufacturer's instructions. Each well was coated freshly with Matrigel (60 µg; BD Bioscience) before the invasion assay. Briefly, 1×10^5^ cells were plated in the top chamber onto the Matrigel coated Membrane (24-well insert; pore size, 8 µm; Corning Costar). Cells were added to the Transwell insert in 0.5 mL of medium containing 1% fetal bovine serum (FBS), which was seated in 750 µL of complete medium (10% FBS) with or without the IKK inhibitor BAY 11-7082 (10 µmol/L). After a 48 h incubation under hypoxic or normoxic conditions, noninvading cells were mechanically removed by a cotton swab. Cells that had migrated through the Matrigel were stained with crystal violet. Cells were counted in five representative microscopic fields (×100 magnification) and photographed.

### Transient transfection and RNA interference

The human pcDNA3.0/HIF-1α (a gift from Max Gassmann PhD, Institute of Veterinary Biochemistry and Molecular Biology, University of Zurich, Zurich, Switzerland) were purified in large scale using the EndoFree Plasmid Maxi kit for transfection. The human pancreatic cancer cells were transiently transfected with an pcDNA3.0/HIF-1α, or empty vector (pcDNA3.0) as control using LipofectamineTM 2000 and Plus reagent in 6 or 12 well plates. The transfections were performed according to the manufacturer's instructions. After transfection, cells were treated as design under normoxic conditions and then harvested for the following experiments.

To knock-down NF-κB p65 subunit, synthesized siRNAs duplex were obtained from Invitrogen. A double-strand siRNA (p65 siRNA), (sense, 5′-GCC CUA UCC CUU UAC GUC A-3′, antisense 5′-UGA CGU AAA GGG AUA GGG C-3′) with two introduced thymidine residues at the 3′ end, which encoded amino acid residues 347 and 353 of the NF-κB p65 subunit was designed to target the NF-κB p65 subunit as described previously [25]. A nonspecific control siRNA (sense, 5′-UUC UCC GAA CGU GUC ACG U-3′; antisense 5′-ACG UGA CAC GUU CGG AGA A-3′) was also designed. BxPC-3 and PANC-1 cells were grown to 50% confluence in 6-well plates, and transfected with the siRNAs in serum-free medium without antibiotic supplements using LipofectamineTM 2000. Cells were allowed to stabilize for 12 h before being used in experiments. Silencing of protein expression was confirmed by Western blot analysis.

### Immunofluorescence staining

After designated treatment, BxPC-3 and PANC-1 cells were fixed with 4% paraformaldehyde for 10 min, permeabilized in 0.5% Triton X-100 for 10 min, and incubated in PBS and 10% goat serum blocking solution for 1 h. Fixed cells were incubated for 2 h with Mouse anti-human-E-cadherin, Rabbit anti-human-Vimentin, in 5% goat serum. Cells were washed and incubated with Goat anti-mouse FITC (green) or Goat anti-rabbit RBITC (red) IgG antibody (ZSGB-BIO Inc., Beijing, China) diluted 1∶100 in blocking buffer for 1 h. Nuclei were stained with DAPI for 0.5 h. Cells were examined with a fluorescent microscope equipped with narrow band-pass excitation filters to individually select for green, red, and blue fluorescence. Cells were observed through a Canon Power shot A640 camera mounted on Fluorescent microscope (Nikon, Japan). Experiment was repeated thrice.

### Electrophoretic mobility shift assay (EMSA)

NF-κB activity was measured by EMSA as we have previously described [25]. The sequences of the oligonucleotides used for the EMSA are as follows: NF-κB, 5′-AGT TGA GGG GAC TTT CCC AGG C-3′ (wild-type) and 5′-AGT TGA GGC GAC TTT CCC AGG C-3′(mutant). EMSA was performed by incubating 10 µg of nuclear protein with Gel Shift Binding buffer and 1 µg of poly (deoxyinosinic-deoxycytidylic acid) at 4°C for 30 min, and then mixed with biotin-labeled oligonucleotide bio-NF-κB probe at room temperature for 20 min, according to the manufacturer's instructions (Viagene, Beijing, China). The resulting DNA-protein complex was separated from free oligonucleotide on a 4% polyacrylamide gel containing 0.25×TBE (Tris/borate/EDTA) buffer. The dried gels were visualized with a CoolImager imaging system (IMGR002), and radioactive bands were quantified using Scion Image software. To check if the observed shifted bands are specific for NF-κB by competition tests using unlabeled wild-type NF-κB or mutant NF-κB probes, nuclear extracts were incubated with 50-fold molar excess of unlabeled probes before the addition of the labeled probes: to a protein extract that displays intense shifted bands, additionally to the labeled NF-κB probe a nonlabeled (“cold”) oligonucleotide is added in excess. If the observed signals are NF-κB specific, those signals should disappear in the presence of the cold NF-κB wild-type competitor but should be unaffected in the presence of the cold “mutant” competitor.

### Western blotting

The methodology has been described previously [18]. Briefly, 5×10^5^ cells were sonicated in RIPA buffer and homogenized. Debris was removed by centrifugation at 12,000 g for 10 min at 4°C. The samples containing 50 µg protein were electrophoresed on polyacrylamide SDS gels, and transferred to polyvinylidene difluoride (PVDF) membranes. The membranes were blocked with 3% BSA, incubated with primary antibodies, and subsequently with alkaline phosphatase-conjugated secondary antibody. They were developed with 5-bromo-4-chloro-3-indolyl phosphate/nitro blue tetrazolium (Tiangen Biotech Co. Ltd., Beijing, China). Blots were also stained with anti-β-actin Ab as internal control for the amounts of target proteins.

### Statistical analysis

The results were expressed as mean values ± standard deviation, and a Student's t test was used to evaluate statistical significance. A value of less than 0.05 (*p*<0.05) was used for statistical significance.
